# Brain-derived neurotrophic factor interplay with oxidative stress: neuropathology approach in potential biomarker of Alzheimer’s disease

**DOI:** 10.1590/1980-5764-DN-2023-0012

**Published:** 2023-12-04

**Authors:** Robert Shen, Christian Ardianto, Celia Celia, Veronika Maria Sidharta, Poppy Kristina Sasmita, Irawan Satriotomo, Yuda Turana

**Affiliations:** 1 Atma Jaya Catholic University of Indonesia, School of Medicine and Health Sciences, Jakarta, Indonesia.; 2 University of Florida, Gainesville, Department of Neurology, Florida, USA.; 3 Satriotomo Foundation, Indonesia Neuroscience Institute, Jakarta, Indonesia.

**Keywords:** Alzheimer Disease, Oxidative Stress, Brain-Derived Neurotrophic Factor, Biomarkers, Antioxidants, Doença de Alzheimer, Estresse Oxidativo, Fator Neurotrófico Derivado do Encéfalo, Encéfalico, Biomarcadores, Antioxidantes

## Abstract

The aging population poses a serious challenge concerning an increased prevalence of Alzheimer’s disease (AD) and its impact on global burden, morbidity, and mortality. Oxidative stress, as a molecular hallmark that causes susceptibility in AD, interplays to other AD-related neuropathology cascades and decreases the expression of central and circulation brain-derived neurotrophic factor (BDNF), an essential neurotrophin that serves as nerve development and survival, and synaptic plasticity in AD. By its significant correlation with the molecular and clinical progression of AD, BDNF can potentially be used as an objectively accurate biomarker for AD diagnosis and progressivity follow-up in future clinical practice. This comprehensive review highlights the oxidative stress interplay with BDNF in AD neuropathology and its potential use as an AD biomarker.

## RESUMO

O envelhecimento da população representa um sério desafio no que diz respeito ao aumento da prevalência da doença de Alzheimer (DA) e o seu impacto na carga, morbidade e mortalidade globais. O estresse oxidativo, como uma marca molecular que causa suscetibilidade na DA, interage com outras cascatas de neuropatologia relacionadas à DA e diminui a expressão do fator neurotrófico encefálico (*brain-derived neurotrophic factor* – BDNF), uma neurotrofina essencial que serve como desenvolvimento e sobrevivência nervosa, e plasticidade sináptica na DA. Pela sua correlação significativa com a progressão molecular e clínica da DA, o BDNF pode potencialmente ser usado como um biomarcador objetivamente preciso para o diagnóstico da DA e acompanhamento da progressividade na prática clínica futura. Esta revisão abrangente destacou a interação do estresse oxidativo com o BDNF na neuropatologia da DA e seu uso potencial como biomarcador da DA.

## INTRODUCTION

Alzheimer’s disease (AD), the foremost irreversible neurological disorder, is the fifth leading cause of death and one of the significant global burdens of diseases; it poses a serious challenge in the aging population that requires research focusing more on the early detection and prevention of AD progression^1–3^. Accurate biomarkers for early AD detection and progression rate monitoring are essential in prevention strategies and assessing therapeutic efficacy. The importance of accelerating research in this regard is because we are pacing with time, along with the increased AD prevalence exponentially with age, and the doubling dementia incidence per year, that projected around ten million additional cases per year, reaching 152 million cases by 2050^4,5^. This comprehensive review is limited to the study of the brain-derived neurotrophic factor (BDNF) as a potential biomarker for AD diagnosis and progression, the complex effects of oxidative stress (OS) in the neuropathology of AD, and the interplay between BDNF, OS, and AD continuum. This study was conducted using extensive research on PubMed, DOAJ, EBSCO, and Google Scholar databases with publications within the last thirty years, prioritizing clinical trials, *in vivo* and *in vitro* studies, randomized controlled trials, and comprehensive review articles. The keywords used for the selection of the pieces were focused on ‘Alzheimer’s disease’, ‘dementia’, ‘cognitive impairment’, ‘aging’, ‘oxidative stress’, ‘reactive oxygen species’, ‘biomarker’, and ‘brain-derived neurotrophic factor’ or ‘BDNF’ and those synonyms. Studies that were irrelevant to this review’s limitation were excluded.

The complexity of AD neuropathology from the genetic, molecular, and anatomic to clinical levels that are influenced by multiple causes, makes important and necessary a paradigm shift toward a multimodal and multispecialty approach in treating AD^6–10^. OS is one of the findings postulated to be involved in the aging process and neurodegenerative diseases, including AD^11^. Aggregation of β-amyloid (Aβ) plaque and neurofibrillary tangles (NFTs) as the main clinical hallmarks of AD was closely associated with increased reactive oxygen species (ROS) production that caused OS as a result of mitochondrial damage and neuronal dysfunction^12,13^. OS is also cited as a molecular hallmark that causes susceptibility in AD and increases the aggregation and production of Aβ and phosphorylation of tau protein leading to a vicious circular process in AD progression^12,14^.

OS decreased the expression and levels of BDNF, the most widely distributed neurotrophin in the brain, that plays an essential role in AD neuropathology, in nerve development and survival, and synaptic plasticity^15–18^. AD patients have significantly lower circulating BDNF levels, especially in the temporal, frontal, and parietal lobes, along with severely reduced BDNF mRNA in the hippocampus and parietal cortex, which leads to cholinergic cell atrophy and dysfunction^19–23^. BDNF can potentially be used as an objectively accurate AD biomarker as it correlates and is directly linked with the cerebral phenomenon of AD, and decreased BDNF levels are also correlated with the degree of cognitive decline and neurological impairment reflecting the AD progressivity^24,25^. Further discussion of BDNF interplay with OS in AD neuropathology and BDNF potential as a diagnostic biomarker and progressivity follow-up will be presented in this review.

### Overview of oxidative stress and its detrimental effects on health

Research on ROS has developed quite rapidly and comprehensively in the last two decades. ROS, which are by-products of aerobic metabolism, play a role in tissue damage in OS conditions and also have an important role in cell signaling pathways, both in various diseases. OS is an imbalance condition of redox state when the amount of pro-oxidants exceeds antioxidants^26,27^. Under certain conditions, the balance of the system in the body, which is maintained by deoxyribonucleic acid (DNA), protein, carbohydrates, and lipids, is damaged by ROS, which causes the disruption of metabolic status, cell growth, and cell development^28^. ROS cause OS by breaking the cellular DNA chain and damaging nitrogenous bases, which results in exposure to hydroxyl radical (^•^OH), one-electron oxidants, singlet oxygen (^1^O_2_^•^), and hypochlorous acid (HOCl)^28,29^. One-electron oxidant was found to potentially decrease the DNA base by order of guanine less reduced than adenine, and both less decreased than cytosine and thymine^29^. OS generated by cell metabolism, an endogenous process or exogenous process by exposome, can be triggered by various causes of tissue damage physically, chemically, or microbially; including infectious conditions, impaired blood vessel perfusion, toxins, radiation, extreme temperature, excessive exercise, and traumatic injury^26,30,31^. Mitochodria is the major source of endogenous ROS, while the exogenous ROS sources are smoking, air pollutants, and solar ultraviolet radiations (UVR), besides pesticides, environmental chemicals, pollutants, redox-active metals, and ionizing radiations from radioactive decay and x-ray photons^32–34^.

ROS, which are sometimes referred to as reactive oxygen metabolites (ROMs) or reactive oxygen intermediates (ROIs), are all reactive species that contain oxygen atoms with unstable and highly reactive properties^35^. ROS are divided into two types, namely oxygen free radicals and non-radicals. Free radicals are every atom that is capable of independent existence and contains more than equal to one unpaired electron in its outer valence area, for example, superoxide, hydroxyl, peroxyl, alkoxyl, and hydroperoxyl^28,35^. In contrast, non-radicals are atoms that do not have unpaired electrons, for example, hydrogen peroxide, hypochlorous acid, ozone, singlet oxygen, and peroxynitrite. ROS can be produced during metabolic processes and the immune system in the human body. The ROS themselves are like a double-edged sword, that is, in addition to the damage they cause at functional concentrations, they have beneficial properties for the body, such as being involved in phagocytosis, apoptosis, necrosis, and protection against pathogens. Actually, upregulation of ROS is the body’s adaptation response to cellular stress due to various physiological disorders; this condition is termed oxidative eustress where the OS is at a low level and the goal is to increase cell resistance as a pre-conditioning for acute stress^26,36^. Whereas the high-level OS, which refers to a pathological condition, is also termed oxidative distress that happens to disrupt redox signaling and molecular oxidative damage^26^. Various studies have found a link of OS to various diseases depending on tissue damage in the related organs ranging from neurodegenerative and metabolic disorders to multi-organ conditions including cancer, aging, and age-related diseases^30,37^. Mild cognitive impairment (MCI) with AD and its severity progression is also linked to OS.

The production of ROS in cells can occur in the mitochondria, peroxisomes, or through misfolded proteins in the nucleus that cause stress on the endoplasmic reticulum^28,38^. Endogenous sub-cellular ROS products involve several enzymes for the generation of ROS, including monoamine oxidase, lipoxygenase, cyclooxygenase, NADPH oxidase, cytochrome p450 monooxygenase, xanthine oxidoreductase, and nitric oxide synthase^28^. Exogenous sources usually form ROS by the Fenton and Haber-Weiss reactions, and these two reactions play a significant role in OS in many neurodegenerative diseases^39–41^. In the Fenton reaction, one of the electrons from molecular oxygen is removed to form superoxide anion; then with superoxide dismutase (SOD) it forms hydrogen peroxide (H_2_O_2_), which reacts with metal transitions (iron/Fe, copper/Cu, zinc/Zn, or aluminum/Al) to form highly ROS, and finally hydroxyl radicals (^•^OH) as output^28,39,40^. H_2_O_2_ which is more stable and permeable to plasma membrane than superoxide, then reacts with superoxide and also forms the hydroxyl radicals (^•^OH) and hydroxyl anion (-OH) — this is the Haber-Weiss reaction^28,40^. H_2_O_2_ through the enzyme myeloperoxidase (MPO) is also able to react with halogen atoms (chlorine/Cl^−^, bromine/Br^−^, and iodine/I^−^) to form hypochlorous acid (HOCl), which is the most reactive and bactericidal ROS, has an important feature as a protective factor against pathogenic invasion, and is closely related to various inflammatory diseases^28,42^. MPO, in a process that irreversibly inactivates through the oxidative burst of neutrophils that occurs in the phagosome, will use H_2_O_2_ for the production of antimicrobial oxidants; this natural chemical signaling molecule in inflammation would jump-start the neutrophils as first responder in immune system against toxins, parasites, bacteria, viruses, and yeast^28,43,44^.

### The role of oxidative stress in neuropathology of Alzheimer’s disease

### Oxidative stress affects aging and Alzheimer’s disease

Aging is an inescapable consequence of entropy that governs the chemical reactions required for life. It is a sequel of time-dependent deterioration of macromolecules with an alteration of our body’s biological system of repair, recycling, and renewal mechanisms^45^. Several things contribute to evolutionary theories of aging, including cellular mechanisms that drive aging, genomic instability, telomere attrition, mitochondrial damage, protein damage, cellular senescence, altered intercellular signaling, dysregulation of the immune system, altered metabolism, and nutrient signaling^45,46^. During life, somatic cells are continually exposed to exogenous pro-oxidants that trigger ROS formation and are capable of causing genomic alterations due to DNA damage; however, it is difficult to determine whether the accumulation of damaged DNA in cells is a result or a consequence of aging itself^45,47^.

Lack of DNA repair mechanisms was found in age-related neurodegenerative disease, and accumulation of DNA damage tends to occur in neurons of people with neurological disorders. Since the study of DNA damage in the early 1990s, it was found that DNA strand breakage in the cerebral cortex of the AD group is twofold higher than in healthy elderly^48^. The mutation in mitochondrial DNA (mtDNA) is also crucial because it affects the work of electron transport chain (ETC) for oxidative phosphorylation. Consequently, it disrupts the respiratory function of cells, causes an abnormal increase in ROS production, and dysregulation of ROS-dependent cellular signaling pathway. This induces abnormal OS, and over lifetime causes metabolic alterations, chronic inflammatory responses, and age-dependent tissue degeneration and dysfunction due to accumulation of cellular damage, respiratory deficiency, and decreasing adenosine triphosphate (ATP), required for cellular functional work^45,49,50^. It should be noted that the frequency of mtDNA mutations is higher than nuclear genome mutations because the mtDNA lacks protective histones and repair enzymes, and mitochondria work in an oxidative microenvironment, so that damage to mitochondrial functionality is more susceptible to triggering OS^50^. In the post-mortem brain sample of AD patients, it was found that there was mitochondrial genomic dysfunction as indicated by the high percentage of cytochrome c oxidase-deficient neurons and the high number of mutations and degraded mtDNA in neurons with ETC disorders^45,51–53^. In addition, a base excision repair (BER) pathway that functions for DNA repair was found defective in the mitochondria of AD patients^45,51–53^.

The occurrence of OS is closely related to the optimal work of mitochondrial cells^13^. In healthy cells, a source of free radical superoxide can be produced in the ETC during mitochondrial activity or generation by NADPH oxidases, which are enzyme complexes in the cell membrane, that is involved in cell signaling and tissue homeostasis^13,54,55^. Superoxide is converted directly to H_2_O_2_ by SOD in the mitochondrial matrix and cytosol to prevent the inactivation of proteins containing iron-sulfur clusters in the mitochondrion^56^. Then the H_2_O_2_ is rapidly converted to water in the glutathione (GSH) redox cycle, including GSH reductase and peroxiredoxins, which are important defense mechanisms in the protection of cell membranes against OS; the purpose is to prevent the continuation of the Fenton reaction in making the most harmful ROS, namely hydroxyl radical, besides, it also reduces the oxidation of lipid molecules^13,57^.

**Figure 1:**
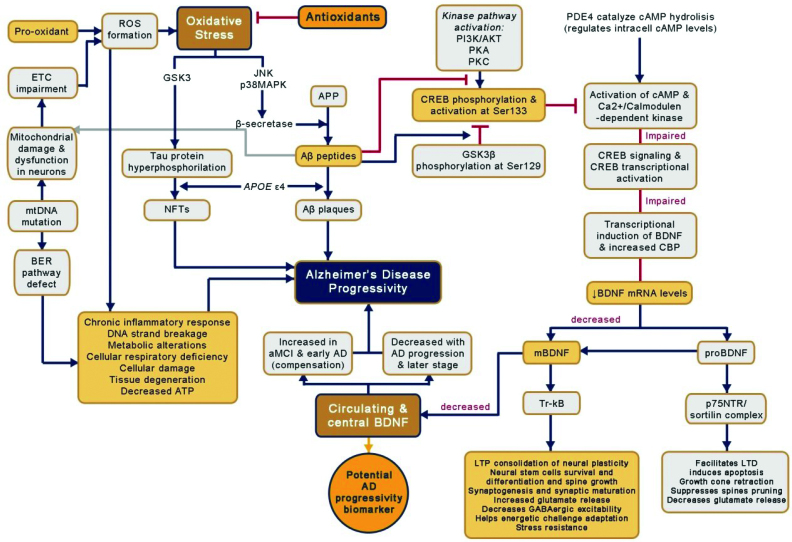
Oxidative stress interplay with brain-derived neurotrophic factors in Alzheimer’s disease neuropathology.

Abbreviations: Aβ, amyloid-β; aMCI, amnestic mild cognitive impairment; APP, amyloid precursor protein; APOE, apolipoprotein E; BER, base excision repair; BDNF, brain-derived neurotrophic factor; cAMP, cyclic adenosine monophosphatase; CBP, CREB-binding protein; CREB, cAMP response element-binding protein; ETC, electron transport chain; GSK3, glycogen synthase kinase 3; JNK, c-jun N-terminal kinase; LTP/D, long term potentiation/depression; mtDNA, mitochondrial DNA; NFTs, non-fibrillary tangles; P13K/AKT, phosphatidylinositol 3-kinase/protein kinase B; p38MAPK, p38 mitogen-activated protein kinase; p75NTR, p75 neurotrophin receptor; PDE4, phosphodiesterase 4; PKA, protein kinase A; PKC, protein kinase C; ROS, reactive oxygen species; Tr-kB, tropomyosin receptor kinase B.

Increased mitochondrial damage and dysfunction in neurons in AD caused by Aβ, that directly bind to mitochondria and interfere with imports of mitochondrial proteins, cause the impairment of ETC function associated with up-regulation of the nitric oxide synthase (NOS) and NADPH oxidase (NOX) genes, and, consequently increases ROS production oxidative stress indexes (OSI) in AD^58,59^. However, like a vicious cycle, OS also increases the generation of Aβ peptides and the formation of NFTs by activating the c-jun N-terminal kinase (JNK) and p38 MAP kinase (MAPK), which then increases β-secretase expression by activating glycogen synthase kinase-3 (GSK3), which causes tau hyperphosphorylation (Figure 1)^60,61^.

### Oxidative stress in Alzheimer’s disease neuropathology

The AD neuropathology is very complex, involving various aspects such as neurodegeneration, chronic inflammation, neurogenesis, disruption of the blood-brain barrier (BBB), vascular homeostasis, impaired cellular signaling, and decreased neurotrophic factors, as well as disorders at the molecular level caused by OS and at the genetic level caused by several genetic variations and mutations, yet aggravated by other chronic and metabolic diseases (Figure 1)^6–10^. The hallmarks of AD neuropathology are the formation and accumulation of highly insoluble densely packed filaments of Aβ plaque extracellularly and NFTs intracellularly in the brain^62,63^. Accumulation of Aβ plaques and NFTs correlated with the progression of memory and cognitive impairment by its cause of neuronal synapse damage, although AD severity is also contributed by multifactorial co-pathology^62,64^.

Aβ plaque is a formation of amyloid fibrils formed from the aggregation of oligomers due to the accumulation of a number of Aβ peptide monomers. The formation of these monomers is due to the amyloidogenic peptides cleavage of extracellular transmembrane proteins of amyloid precursor protein (APP) by β-secretase and γ-secretase, which in physiological processing by α-secretase and γ-secretase in the non-amyloidogenic peptides cleavage pathway is thought to have function for modulating a cell and neurite growth and survival^63,65–68^. On the other hand, the nerve axon that contained an abundance of microtubule-associated protein (MAP) tau has a role in promoting the stabilization of microtubules (MTs) of its six tau isoforms that compose the MT-binding domain^69,70^. The condition of tau hyperphosphorylation caused by indirect events (Aβ mediated neurotoxicity, OS, and chronic inflammation) as in AD, and the direct events (aberrant activation of tau kinases, downregulation of phosphatases, mutations, and covalent modifications of tau), will cause a loss of binding between MAP tau and MTs and trigger the formation of NFTs aggregation composed of misfolded tau protein deposits in neurons or glia cells, also called tauopathies, that would cause neurotoxicity and compromised axonal transport^69,71–73^. Like a vicious cycle, Aβ can trigger the tau protein conversion to a toxic state, and as a feedback loop, it will also enhance the Aβ itself^62^.

Although cerebral oxidative damage is a part of aging and neurodegenerative diseases, OS and inflammatory processes are also environmentally driven risk factors that were found to interact with apolipoprotein E (ApoE)-encoding, the *APOE*-gene that plays a significant role in the neuropathology course of AD and leads to a susceptibility of AD development and progression^74^. In AD, Aβ which was transported into cells contributing to ROS, which then induced the cytokine response interleukin-6 (IL-6) and simultaneously with activation of NF-kB in the nucleus will activate the expression of the *APOE*-gene^13,74,75^. Neurochemically, ApoE was co-localized with AD neuropathological lesions, where plaques and tangles were deposited, and an increase of ApoE mRNA in astrocytes was found in the hippocampus and other regions of the brain that had degenerated neuron cell bodies or synaptic remodeling; this event indicates the occurrence of lipid uptake in the neurodegeneration process of AD^74,76^.

The *APOE*-gene is one of the strongest genetic risk loci associated with late-onset AD (LOAD), increasing the risk of events 3 to 15-fold^77,78^. ApoE is a 299-amino-acid protein polymorphic that is synthesized and secreted mainly in the liver and brain by astrocytes, oligodendrocytes, activated microglia, and ependymal layer cells, and a lower level of expression was also found in central nervous system (CNS) neurons; functionally as cholesterol transporter protein among the various cells including in the brain^74,79,80^. Among three alleles of *APOE-*gene (ε2, ε3, and ε4) that encode three isoform proteins, the ε4 allele plays a major role as a susceptibility factor of AD development^79,81^. Histopathological studies also showed that the ε4 allele was associated with the amount of neuritic plaque and NFTs, suggesting its role in mediating AD neuropathology accumulation in line with clinical changes^74^. *APOE* ε4 has neurotoxic properties, promotes fibrillogenesis, and has been found to be associated with increased risk and accelerated age-onset of AD^82^. The interaction circle of Aβ, ApoE, cholesterol, and APP will form a cascade of a series of events in the pathogenesis of AD along with molecular changes of OS (Figure 1).

### Brain-derived neurotrophic factor interplay with oxidative stress and its potential as Alzheimer’s disease biomarker

### Overview of brain-derived neurotrophic factor in neurodegenerative diseases

**Figure 2:**
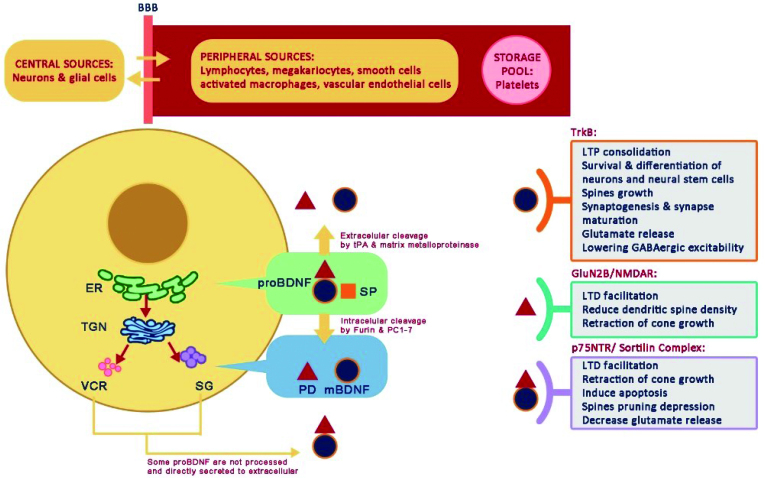
Brain-derived neurotrophic factor synthesis, functions and sources.

Abbreviations: BBB, blood-brain barrier; BDNF, brain-derived neurotrophic factor; ER, endoplasmic reticulum; GABAergic, gamma-aminobutyric acidergic); LTP/D, long term potentiation/depression; mBDNF, mature-BDNF; PC 1-7, proprotein convertase 1 to 7; p75NTR/Sortilin Complex, p75 neurotrophin receptor complexed with sortilin; PD, pro-domain; SG, secretory granules; SP, signal peptide; TGN, trans-Golgi network; tPA, tissue plasminogen activator; TrkB, tropomyosin-related receptor kinase B; GluN2B/NMDAR, GluN2B containing N-methyl-D-aspartate/NMDA receptors; VCR, vesicles for consecutive release.

BDNF is a neurotrophin synthesized by neurons and has a key role in the development and maturation of the nervous system, including neuronal survival, restoration, and differentiation, besides synaptic function, axonal and dendritic growth, and energy requirements in carrying out nerve functions^24,83,84^. BDNF was initially synthesized as a propeptide (proBDNF) in the lumen of the endoplasmic reticulum, together with the signal peptide. The proBDNF inside the trans-Golgi network (TGN) will undergo a cleavage process dividing the active peptides of mature-BDNF (mBDNF; pre-domain) from the pro-domains site by the works of proteolytic enzyme intracellular proprotein convertases (PCs) — furin and PC1 to PC7 — that can pass through two types of vesicles in TGN, namely through vesicles for consecutive release (VCR) of the consecutive secretion pathway or through secretory granules (SG) of the regulated secretion pathway; meanwhile, if it reaches the extracellular matrix before the intracellular cleavage, the proBDNF is cleaved by extracellular proteases, plasmin (tissue plasminogen activator/tPA), and matrix metalloproteases (Figure 2)^15,85^.

Not all proBDNF is transformed into mBDNF, some are not processed under proteolytic cleavage and secreted exocytosis by VCR or SG directly to the extracellular^24^. The mBDNF binds to the high-affinity tropomyosin-related receptor kinase B (TrkB) and fosters several benefits as the consolidation of long-term potentiation (LTP) involved in neural plasticity; it triggers neural stem cells survival and differentiation and spine growth; helps synaptogenesis and synaptic maturation; increases glutamate release; decreases neuronal GABAergic (gamma-aminobutyric acidergic) excitability; and helps energetic challenge adaptation and stress resistance^24,86^. In addition, the pro-domains that have been separated from mBDNF can be activated by binding to GluN2B containing N-methyl-D-aspartate receptors (NMDAR), triggering a reduction in dendritic spine density and growth cone retraction, and facilitating long-term depression (LTD). The proBDNF, which is not converted into mBDNF, also facilitates LTD by binding to the p75NTR (p75 neurotrophin receptor) and sortilin complexes, which induces apoptosis, growth cone retraction, suppresses spine pruning, and decreases glutamate release^24,86^.

Je et al. proposed proBDNF and mBDNF as reward and punishment signaling models, respectively, for synaptic elimination of neuromuscular junctions (NMJs) based on *in vivo* experiments by inhibiting proteolytic conversion to mBDNF. Thus, the proBDNF would accelerate the presynaptic elimination of axon terminals through p75NTR activation, and, in contrast, when p75NTR and sortilin signaling was inhibited, it would attenuate and delay the synaptic elimination process^87^. Even elevated plasma levels of proBDNF and excessive proBDNF in the brain can exhibit stereotypical behavior, impaired social interaction, hyperactivity, and increased stress response^88^. The role of BDNF in neurogenesis and synaptic plasticity also illustrates the susceptibility that arises when the production of mBDNF in carrying out its functions is inadequate, and this is reflected in a significant decrease in BDNF levels in neurological diseases such as AD and other types of dementia, Parkinson’s disease, epilepsy, Huntington’s disease, as well as neurobehavior disorders such as schizophrenia, autism, depression, and social avoidance^84,89,90^.

The condition of impaired cognitive function and memory formation in AD is not far from the area of the brain that supports its function; the interconnection between the entorhinal cortex (EC) and the hippocampus, and BDNF also influences the processes within this learning pathway. In these two areas, there is information flow that forms organized circuit loops, corticohippocampal and intrahippocampal connections that play a role in the learning process; and brain synaptic plasticity has a role in learning that includes sensory experiences and adaptive processes to spatial, episodic, social, and contextual memory^91,92^. These neoclassical pathways of the corticohippocampal circuit start from the entry of glutamatergic input from the superficial EC in the area that carries nonspatial sensory information (lateral EC/LEC) and spatial information (medial EC/MEC)^91,92^. Grid cells on EC in layers II (LII) and layers III (LIII) then carry information for processing in the hippocampus on several parallel circuits: CA1 pyramidal neurons via trisynaptic paths (EC LII to the dentate gyrus, then to CA3 and towards CA1) or monosynaptic paths (EC LIII to CA1); CA1 also gets direct monosynaptic projection from MEC LII; besides, CA2 gets direct input from LII MEC and LEC before going to the dendritic domains of stratum oriens/radiatum CA1 which overlaps with the input from CA3 to CA1; to complete the circuit loop, the hippocampus performs a back-projection to the deep EC^91,93,94^.

BDNF performs LTP modulation, which is useful in preserving synaptic plasticity function in the Schaffer collateral terminals – CA1 area of the hippocampus^95–97^. Although previously found that postsynaptic and interneuronal transmissions have the potential to be BDNF locus of action, a later study observed that BDNF exclusively enhances transmitter release on TrkB receptors, not p75NTR, on presynaptic CA3 afferent neurons or interneurons for synaptic LTP modulation in the CA1 region^95–97^. Thus, BDNF signaling is not directly involved in the biochemical changes of LTP in the postsynaptic neurons but by modulation of repetitive exocytotic events presynaptically that indirectly modify LTP response postsynaptically^95–97^. In contrast to LTP, the LTD process in the hippocampus is due to activation of proBDNF-p75NTR signaling localized in dendritic spines and CA1 afferent terminals. Furthermore, LTD activity is uniquely due to decreased expression of NMDAR subunit 2B, so deletion of the p75NTR receptor selectively disrupts NMDAR-dependent LTD without affecting LTP synaptic plasticity, both proBDNF and BDNF acting bidirectionally^98^.

### How oxidative stress affects brain-derived neurotrophic factor and the factor as a potential biomarker of Alzheimer’s disease

BDNF expressed in the CNS and blood has been found to be involved in the neuropathology of various neurodegenerative diseases, including AD. An imbalance or lack of mBDNF causes impaired neuronal plasticity that underlies a hypothesis of the strategy to optimize mBDNF transformation as a prospective AD therapy target^15,16^. BDNF stimulates the non-amyloidogenic pathway of APP as a protective factor for AD, but OS leads to decreased BDNF levels and, in turn, leads to increased Aβ, which worsens AD progression^99,100^.

BDNF was found to potentially protect against neurotoxicity induced by Aβ and restore the nerve alteration induced by Aβ 1-42^101,102^. Vice versa, downregulation of BDNF expression caused by Aβ lead to cognitive dysfunction and loss of memory, as BDNF is involved in brain areas of the hippocampus, cortical and basal forebrain function for learning, memory, and higher cognitive function^101–105^. Aβ plaques are involved in impaired BDNF synthesis and transduction of neurotransmitters, leading to blockage of synapse and accelerated nerve degeneration that is also underlying AD etiology^89,106^. Quantification of BDNF mRNA levels in AD brain tissue showed a 3.4-fold decrease compared to the control group^23^. ProBDNF buildup in mouse models indicates decreased brain volume, reduced dendritic arborization, impaired synaptic transmission, and neuronal plasticity^88^. Lu et al. described the activity of the mature neurotrophin and its precursor proBDNF at their respective receptors as “yin and yang” action as they play opposite roles in modulating neuronal synaptic plasticity and survival^107^.

Increased BDNF levels are correlated with an antioxidant defense mechanism against OS^108^. OS roles in AD neuropathology lead to decreased BDNF levels by suppressing and reducing cAMP response element-binding (CREB) expression and its phosphorylated-CREB (pCREB) content, increased nuclear factor-kappa B (NF-kB) DNA-binding activity, and energy depletion (Figure 1)^17,18^. CREB is a major mediator and regulator of the BDNF-induced gene expression response through the regulation of its transcription by the binding of pCREB to a specific sequence in the BDNF promoter^109,110^. CREB as a major neurotrophin response regulator in mature neurons can trigger neurotrophins to induce the expression of regulatory regions in CREB-regulated genes that mediate the long-lasting effects of neurotrophin activity on synaptic function. Through this CREB-dependent gene expression, BDNF can influence and consolidate synaptic strength^109^. From the genetic perspective, the presence of BDNF genetic polymorphism at codon 66, the Met66 allele, was also significantly associated with cognitive impairment as MCI progressed to AD and interacted with ApoE 4^111,112^.

By the fact that OS interplays with BDNF in neuropathology and progression of AD, BDNF levels are a potential biomarker for diagnosis and progressivity follow-up. No molecule has been proven to be conclusive for AD diagnosis at the pre-symptomatic stage until today^24^. However, there are several possible molecular biomarkers of AD associated with its progressivity: BDNF and Pittsburgh compound B positron emission tomography (PET) in the brain; Aβ1-42, neurogranin, and total and phosphorylated tau protein in cerebrospinal fluid (CSF); and examination of miR-107 mRNA, plasma neurofilament light, platelet amyloid precursor protein isoform ratio, lipid peroxidation products, and vascular cell adhesion molecule-1 in blood^24^. Based on the biomarker effectiveness for an early AD diagnosis, it is necessary to consider the ease of examination, the availability of extensive facilities, and invasiveness minimization. Thus, the use of circulating BDNF through peripheral blood fulfills the effectiveness of this biomarker; however, further validation in properly wide longitudinal studies comparing to current consolidated biomarkers of AD should be conducted to include BDNF as a valid molecular biomarker. In the future, BDNF may possibly be added as a newly available biomarker to the amyloid-tau-neurodegeneration (ATN) system developed by Jack Jr et al. in the National Institute on Aging and Alzheimer’s Association (NIA-AA) Research Framework that is widely used to diagnose AD that, not only focuses on cognitive staging, but also on any biomarkers changes in the AD continuum^113^. The new AD biomarker could contribute to defining AD severity in the AT(N) system, as N covers up all neurodegenerative or neuronal injury biomarkers. B sides, in the NIA-AA framework, it is stated that new biomarker groups could be added when it is available, without ruling out A (Aβ) and T (pathologic tau) as the unique neuropathologic biomarker to diagnose AD and exclude other causes of dementia. Circulating BDNF levels reflect the progression of AD staging as an early compensatory increase in amnestic-MCI (aMCI) and early-stage AD, followed by a decrease in the late-stage AD^24,114,115^. The decrease in BDNF levels may also be associated with cognitive deterioration in healthy elderly; however, a decrease in BDNF levels in AD was significantly lower^89,116,117^. In addition to the usefulness of circulating BDNF as an AD biomarker, Li et al. also show that central BDNF levels from CSF can be an independent predictor to follow the progression of aMCI to AD and signs of cognitive decline^118^.

BDNF is mainly produced by neurons and glial cells in the CNS; however, BDNF can also be synthesized from peripheral sources by vascular endothelial cells, lymphocytes, smooth cells, and activated macrophages, while platelets function as the main storage pool for BDNF^24,119,120^. Although studies on animals and humans have shown a correlation between blood BDNF levels, brain BDNF levels, and the cerebral phenomenon, further research is still needed to determine whether blood BDNF levels accurately and precisely reflect their levels in the brain^24,121^. There is also a difficulty in choosing the use of circulating BDNF — between serum or plasma BDNF — because different results may be obtained from these two matrices; BDNF serum levels are 200 times higher than BDNF plasma levels because they reflect the amount of stored BDNF in circulating platelets released during clotting^24,122^. However, both can also be important biomarkers because serum levels represent a long-term storage pool, whereas plasma levels represent bioactive forms^24^.

**Figure 3:**
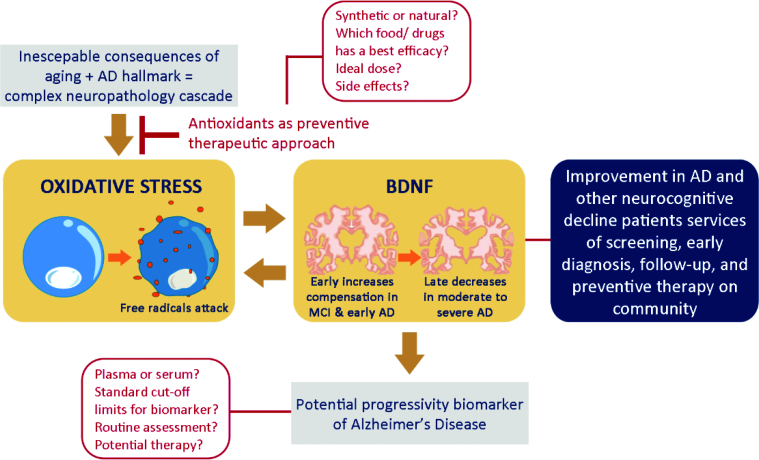
Brain-derived neurotrophic factor as Alzheimer’s disease biomarker and its interplay with oxidative stress and anti-oxidants.

BDNF levels used as a biomarker for AD diagnosis and progressivity follow-up might have a limitation in its specificity because changes in BDNF, as previously described, also occur in several other neurodegenerative and neurobehavior diseases, although not as significant as in AD. OS and all AD-related neuropathology cascades could either influence or be influenced by BDNF, and changes in one system will affect other systems, all of which are involved in the neurodegenerative cascade of AD^111^. Further research is needed regarding the use of BDNF as an AD biomarker, including (Figure 3):

- The diagnostic value and its standard cut-off for each AD stage;
- The best circulation of BDNF, whether plasma or serum;
- The limitation compared to the current gold-standard for diagnosis,
- The possibility for routine assessment to define AD progression; and
- The potential of its use for target therapy.

As an antioxidant happens to be a natural counter for OS, its use as a preventive, therapeutic approach might also be considered to increase BDNF levels. Simple approaches, such as diet modification, can be taken to avoid dietary pro-oxidants and increase natural antioxidant consumption. A study found that a chronic exposure to pro-oxidant substances (e.g., organophosphates in agriculture products) can trigger OS that significantly reduces mRNA expression and protein levels of BDNF^123^. High-fat diet consumption also triggers oxidative damage that reduces BDNF protein and mRNA levels and their downstream effectors (synapsin I and CREB)^18^. The provision of antioxidants can significantly prevent these effects and perform reverse protein oxidation events, and normalizing BDNF levels results in improvements in synaptic plasticity and cognitive function^18^. An experimental study by Handajani et al. also found the benefit of natural antioxidants by consuming 100 grams of Tempeh (soy fermented with *Rhizopus fungi*) per day for six months, which increased the global cognitive scores of MCI elderly, higher than the control^124^.

The essential studies on BDNF roles in AD (Table 1)^6,115,116,125–139^ and the potential therapeutic roles of antioxidants against AD which were registered in the ClinicalTrials.gov database in phase 3 (Table 2)^140–144^ are resumed in the table below. There have been limited validated clinical trials regarding the use of antioxidants against AD that were registered from phase 3 up to now. This study did not include phase 1 and phase 2 clinical trials.

**Table 1:** Study on brain-derived neurotrophic factor and oxidative stress roles in Alzheimer’s disease by year order.

Reference	Size (n) and Design	Diseases	Key findings
Yasutake et al.^125^	60 AD, 60 VaD, 33 healthy elderly controls. Cross-sectional.	AD and VaD	Serum BDNF levels are significantly lower in AD groups than in VaD and controls group. BDNF plays pathological roles in AD, but further investigation is needed.
Laske et al.^126^	15 early stages AD, 15 severe stages AD, 10 healthy elderly controls. Cross-sectional.	AD	Serum BDNF levels increased in the early stages of AD, reflecting the compensatory mechanism of early neurodegeneration, while in time, they will decrease, correlating with the severity of AD because of trophic support lacking and increasing Aβ plaque accumulation contributing to specific progressive degeneration in the affected brain region. BDNF in both groups is not detected in CSF because its level is below the detection limit. Further investigation is needed to define BDNF as an AD clinical diagnosis marker and for therapeutic monitoring.
Laske et al.^127^	27 AD, 9 NPH, 29 healthy elderly controls. Cross-sectional.	AD and NPH	Significant decreases in BDNF serum levels in AD and NPH patients compared to healthy controls, reflecting a lack of trophic support and progressive neurodegeneration. BDNF serum levels did not correlate with MMSE, age, and CSF levels. No significant differences in BDNF levels in CSF in AD, NPH, and controls because of its low concentration. Further investigation is needed to explain the reasons for blood BDNF level reduction between AD and NPH patients.
Leyhe et al.^128^	19 AD, 20 healthy elderly controls. Prospective cohort study.	AD	Serum BDNF levels were significantly decreased in the AD group before AChE-inhibitors treatment (10 mg Donepezil per day) and significantly increased after 15 months of treatment with no more significant difference from the control group. Down-regulation of BDNF begins with the first clinical symptoms and becomes persistent in the AD continuum, while up-regulation of BDNF happens along with the neuroprotective effect of AChE-inhibitor.
Angelucci et al.^115^	86 AD, 54 aMCI and MCI-MD, 27 healthy elderly controls. Cross-sectional.	aMCI, MCI-MD, mild AD, and moderate-sever AD	BDNF has a potential role as a biomarker during the AD course, shown by an upregulation of BDNF serum levels that significantly increased in MCI and AD compared to healthy groups. However, the BDNF levels among aMCI, MCI-MD, mild AD, and moderate-severe AD are insignificantly different. Upregulation of BDNF is an early compensatory against neurodegeneration.
Forlenza et al.^116^	30 AD, 71 MCI, 59 healthy elderly controls. Prospective cohort study.	AD and MCI	Decreased serum BDNF levels, along with a continuum of MCI to AD, indicate a reduced BDNF systemic availability that plays a role in the neurodegenerative process. The presence of the Met-BDNF allele and the APOE ε4 gene predict a worsened cognitive outcome in MCI patients.
O’Bryant et al.^129^	198 AD, 291 controls. Prospective cohort study.	AD	Serum BDNF levels increased in the AD group associated with lower neuropsychological function on visual and verbal memory. Still, they had no significant difference in BDNF levels compared to the control group.
Sonali et al.^130^	63 AD, 15 aMCI, 63 healthy elderly controls. Cross-sectional.	AD and aMCI	Serum BDNF levels were insignificantly higher in the AD group than in the aMCI group controls. BDNF was insignificantly different in genotype and allele distribution between the three groups and did not significantly affect MMSE score in AD and aMCI. Further investigation is needed to examine the potential of BDNF as an AD biomarker.
Ventriglia et al.^131^	266 AD, 28 FTD, 40 LBD, 91 VaD, 30 PD, 169 healthy controls. Cohort retrospective and cross-sectional.	AD, FTD, LBD, VaD, and idiopathic PD.	BDNF levels decreased in several cognitive disorders as a non-specific marker for neurodegeneration. The use of psychotropic drugs during BDNF studies could create a confounding effect, therefore, needs to be controlled before BDNF analysis. BDNF can potentially be used as a new therapeutic target because it involves dementia and PD neuropathology.
Faria et al.^132^	50 AD, 37 MC, 56 healthy elderly controls. Cross-sectional.	AD and MCI	AD patients showed a higher peripheral BDNF level reflecting a compensatory mechanism toward early neurodegeneration and related to immune cell activation (higher sTNFR1 and sICAM-1). Both peripheral BDNF and inflammatory markers are potentials to be an additional tool to differentiate cognitive impairment degrees.
Turana et al.^6^	51 aMCI, 58 healthy elderly controls. Cross-sectional.	aMCI	Low plasma BDNF levels and the presence of the APOE ε4 gene improve the diagnostic value of aMCI diagnostic tool combination using pupillary response and olfactory test.
Liu et al.^133^	110 AD, 120 healthy controls. Cross-sectional.	AD	Significantly lower serum BDNF levels in the AD group suggest an insufficient neurotrophic supply. BDNF levels were also significantly lower among all subjects in the AD group with APOE ε4 gene. BDNF possibly interacted with APOE ε4 and co-effects with MMSE scores. BDNF potential contributed to the molecular mechanism of the AD continuum.
Passaro et al.^134^	44 late onset AD, 50 VaD, 23 CVD not dementia, 47 healthy controls. Cross-sectional.	AD, VaD, CVD not dementia	Plasma BDNF level in dementia (AD and VaD) groups affected by diabetes is the lowest among all subjects. BDNF levels affected by dementia synergically with diabetes status.
Ng et al.^135^	2,067 AD and healthy control. Systematic review and meta-analysis.	AD	Serum BDNF levels were significantly lower in AD (excluding MCI) group than in controls with significant heterogenicity caused by age and MMSE scores as significant moderators during meta-regression analysis. Change in peripheral BDNF is only detected at the late stage of the AD continuum. Further investigation is needed, including molecular mechanisms, interventional trials, and BDNF potential use as an AD biomarker.
Mizoguchi et al.^136^	256 elderlies with independent daily life without dementia. Cross-sectional observational study (2010 to 2016).	Memory impairment	Lower serum BDNF levels, older ages, lower physical activity, and hippocampal atrophy were associated with memory impairment independently. Impaired BDNF function (excluding proBDNF) combined with lower physical activity and hippocampal atrophy is associated with age-related memory impairment; therefore, BDNF is potentially used as a therapeutic target to prevent dementia.
Mori et al.^137^	23 AD, 22 MCI, 21 healthy controls. Cross sectional.	AD and MCI	MCI group had significantly lower serum BDNF levels compared to the control group. AD group had a downward trend of serum BDNF levels than control group, but was not statistically significant. BDNF levels have a positive correlation with Aβ42 levels in CSF. The decreased serum BDNF levels could potentially be used as an AD early detection and progression biomarker.
Ng et al.^138^	40 aMCI and non-aMCI, 56 healthy controls. Case-control.	aMCI, and non-aMCI	Plasma BDNF levels significantly increased in all MCI subjects with good discriminative accuracy as an early compensatory mechanism in preclinical dementia. BDNF is also correlated with neurotrophic and inflammation because it positively correlates with plasma high-sensitivity C-reactive protein.
Perkovic et al.^139^	295 AD, 209 MCI.	AD and MCI	The increase in plasma BDNF levels in AD patients more significantly than in the MCI group might be due to counteracting mechanisms in the early-middle stage of neurodegeneration.

Abbreviations: AChE, acetylcholinesterase; AD, Alzheimer’s disease; aMCI, amnestic mild cognitive impairment; BDNF, brain-derived neurotrophic factor; CSF, cerebrospinal fluid; CVD, cerebrovascular disease not dementia; FTD, frontotemporal dementia; LBD, Lewy body dementia; MCI, mild cognitive impairment; MCI-MD, multidomain mild cognitive impairment; MMSE, Mini-Mental State Examination; NPH, normal pressure hydrocephalus; PD, Parkinson’s disease; proBDNF, propeptide brain-derived neurotrophic factor; sICAM-1, Soluble intercellular adhesion molecule-1; sTNFR1, Soluble tumor necrosis factor receptor-1; VaD, vascular dementia.

**Table 2:** Phase 3 clinical trials on antioxidants interventions in Alzheimer’s disease registered in ClinicalTrials.gov.

Clinical trial official title, NCT ID and access	Participant number and interventions	Diseases and outcome measures	Publication outcome - key findings	
“A Single Center, Multi-site, Randomized, Double-blind, Placebo-controlled Trial of Resveratrol with Glucose and Malate (RGM) to Slow the Progression of Alzheimer’s Disease”	27 participants.	AD	Zhu et al.^140^ Low-dose of oral resveratrol at 12 months showed less deterioration of neurocognitive scores in the AD group than controls. It is safe and well tolerated, but further larger studies are needed to determine its benefit in interpreting clinical outcomes.	
	Dietary Supplement: Resveratrol with Glucose, and Malate (delivered in grape juice)			
		Primary: ADAScog		
NCT00678431 (https://clinicaltrials.gov/show/NCT00678431) “A Randomized, Double-Blind, Placebo-Controlled Trial of Vitamin E and Donepezil HCL (Aricept) to Delay Clinical Progression from Mild Cognitive Impairment (MCI) to Alzheimer’s Disease (AD)”		Secondary: CGIC		
	Dietary Supplement:Liquid placebo			
	720 participants.	AD	Petersen et al.^141^	
		Primary:NINCDS-ADRDA Alzheimer’s Criteria	No significant difference in probable progression to AD in the group given with vitamin E than controls. No benefit of vitamin E in MCI patients.	
	Drug: Vitamin E Drug: Donepezil			
NCT00000173 (https://clinicaltrials.gov/show/NCT00000173)		Secondary: CDR		
“Sunphenon EGCg (Epigallocatechin-Gallate) in the Early Stage of Alzheimer’s Disease”	21 participants.	AD	No results published/posted	
		Primary:ADAS-COG		
	Drug:Epigallocatechin-Gallatea Drug: Placebo	Secondary: Safety and tolerability of the verum; MMSE; Time to hospitalization and Time to death related to AD; Brain atrophy assessed by brain MRI; Baseline-ADAS-COG and Baseline-MMSE as covariates; CIBIC+ and WHOQOL-Bref; Trail Making Test and MVGT		
NCT00951834 (https://clinicaltrials.gov/show/NCT00951834)				
“Study of Melatonin: Sleep Problems in Alzheimer’s Disease”	157 participants	AD and Dyssomnias.	Singer et al.^142^	
		Primary: Change of nocturnal sleep time from baseline to the end of treatment phase		
			Melatonin is not effective a soporific agent in AD group based on actigraphy showed by no statistically significant objective sleep measures difference in intervention and control group.	
NCT00000171(https://clinicaltrials.gov/show/NCT00000171)	Drug: MelatoninDrug: Placebo	Secondary: Time awake after sleep onset, sleep latency, sleep efficiency, daytime agitation, changes in cognition, relative effectiveness of high and low dose melatonin		
“A Randomized, Clinical Trial of Vitamin E and Memantine in Alzheimer’s Disease (TEAM-AD)”	613 participants.	AD	Dysken et al.^143,144^	
			Alpha-tocopherol (Vitamin E) is beneficial in mild and moderate AD by slowing a functional decline and lowering caregiver burden. The differences in the group taking memantine and memantine plus alpha-tocopherol were insignificant.	
	Drug: dl-alpha-tocopherol (Vitamin E)Drug: Memantine (Namenda (R))	Primary: ADCS/ADL		
NCT00235716(https://clinicaltrials.gov/show/NCT00235716)				
		Secondary: MMSE, ADAS-COG, NPI, CAS, Dependence Scale		
“Multicenter Vitamin E Trial in Aging Persons with Down Syndrome”	350 participants.	AD and down-syndrome.		
NCT01594346 (https://clinicaltrials.gov/show/NCT01594346)	Drug: Alpha-tocopherol(Vitamin E)Drug: Sugar pill		No results published/posted	
		Primary: BPT		

Abbreviations: ADAS-COG, Alzheimer’s Disease Assessment Scale-Cognitive; ADCS/ADL, Alzheimer’s Disease Cooperative Study/Activities of Daily Living; BPT, Brief Praxis Test; CAS, Caregiver Activity Survey; CDR, Clinical Dementia Rating Scale; CGIC, Clinical Global Impressions of Change; CIBIC+, Clinician Interview-Based Impression of Change Plus; NINCDS-ADRDA, National Institute of Neurological and Communicative Disorders and Stroke and the Alzheimer’s Disease and Related Disorders Association; NPI, Neuropsychiatric Inventory; MMSE, Mini-Mental State Examination; MVGT, Münchner Verbaler Gedächtnistest; WHOQOL, World Health Organization Quality-of-Life Scale.

In conclusion, OS is an inescapable consequence of aging, along with other AD hallmarks, forming a complex neuropathology cascade of AD. OS affects brain and blood BDNF levels that follow AD progression, and the antioxidant therapy approach may slow its progression. The use of circulating BDNF levels can be a potential molecular biomarker for the diagnosis of AD and monitoring of its progression upon the given prevention and therapy. Further research must be done to define the diagnostic value of BDNF as an AD biomarker for its application in clinical practice.
